# Postoperative pancreatic fistula after gastrectomy for gastric cancer

**DOI:** 10.1002/ags3.12398

**Published:** 2020-09-21

**Authors:** Marie Washio, Keishi Yamashita, Masahiro Niihara, Kei Hosoda, Naoki Hiki

**Affiliations:** ^1^ Department of Upper Gastrointestinal Surgery Kitasato University School of Medicine Sagamihara Japan; ^2^ Division of Advanced Surgical Oncology Department of Research and Development Center for New Medical Frontiers Kitasato University School of Medicine Sagamihara Japan

**Keywords:** gastrectomy, gastric cancer, pancreatic fistula

## Abstract

Postoperative pancreatic fistula is one of the most severe complications after gastric cancer surgery, and can cause critical patient conditions leading to surgery‐related death. Fortunately, the incidence of postoperative pancreatic fistula after gastrectomy seems to be decreasing with changes in operative procedures. The rate was reported at about 30% after open gastrectomy with Appleby's method in 1997, but lately has improved below 1% for robotic gastrectomy in 2019. For the diagnosis of postoperative pancreatic fistula, drain amylase concentration has been demonstrated to be beneficial and some reports have proposed the optimal cut‐off values of drain amylase to predict major postoperative pancreatic fistula. There have been many reports identifying risk factors for postoperative pancreatic fistula, including overweight patients, pancreatic anatomy, blunt trauma from compression of the pancreas, and thermal injuries caused by the continuous use of energy devices. And importantly, laparoscopic gastrectomy has been shown to be more often associated with postoperative pancreatic fistula than open gastrectomy in the prospective national clinical database in Japan. Hence, further sophistication of surgical techniques to reduce pancreas compression would have great promise in reducing postoperative pancreatic fistula after laparoscopic gastrectomy.

## Graphical abstract

Postoperative pancreatic fistula is recently decreasing with great advancement of surgical technique.

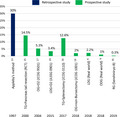

## 1 INTRODUCTION

Gastric cancer remains one of the most important causes of cancer‐related death in the world.^1^ Surgical resection is the recommended treatment for curable gastric cancer, and randomized controlled trials (RCTs) in Asian^2^ and Western countries^3^, ^4^ have demonstrated that extended (D2 or beyond) lymphadenectomy significantly improves patient survival as compared to limited (D1) lymphadenectomy, and D2 gastrectomy could be designated as the standard treatment option for advanced gastric cancer.

Since Kitano et al first demonstrated laparoscopic gastrectomy (LG) as a less invasive alternative to conventional open gastrectomy (OG) for early gastric cancer (EGC) in 1994,^5^ LG for EGC has widely prevailed, especially in Japan and Korea.^6^, ^7^ On the other hand, postoperative pancreatic fistula is an emerging concern for LG in comparison to open surgery, where the real‐world incidence of postoperative pancreatic fistula in prospective cohort studies has been shown to be 1% in open surgery vs 2% in laparoscopic distal gastrectomy (LDG; *P* = 0.04).^8^, ^9^

The mainstays of postoperative pancreatic fistula treatment are reliable drainage of retained pancreatic juice and infection control. If there remains a defective drainage area, it is necessary to adjust the drainage tube position, and in some cases computed tomography‐guided puncture is recommended. Sometimes drainage surgery may even be necessary if drainage is inadequate. In the past, it was reported that continuous intraperitoneal lavage was effective,^10^ and some papers reported that octreotide administration was also effective.^11^, ^12^ Nevertheless, their effectiveness has not be investigated by an RCT. Postoperative pancreatic fistula can cause critical patient conditions, including sepsis or the rupture of pseudoaneurysms, which could eventually lead to surgery‐related death.

Thus, it is important to reduce the risk of postoperative pancreatic fistula after gastrectomy. In this article, we will review the current understanding of the trend in postoperative pancreatic fistula rates, along with its diagnosis, prediction, and prevention in gastric cancer surgery.

## 2 INCIDENCE OF POSTOPERATIVE PANCREATIC FISTULA AFTER GASTRECTOMY FOR GASTRIC CANCER

### 2.1 Open gastrectomy

**Figure 1:**
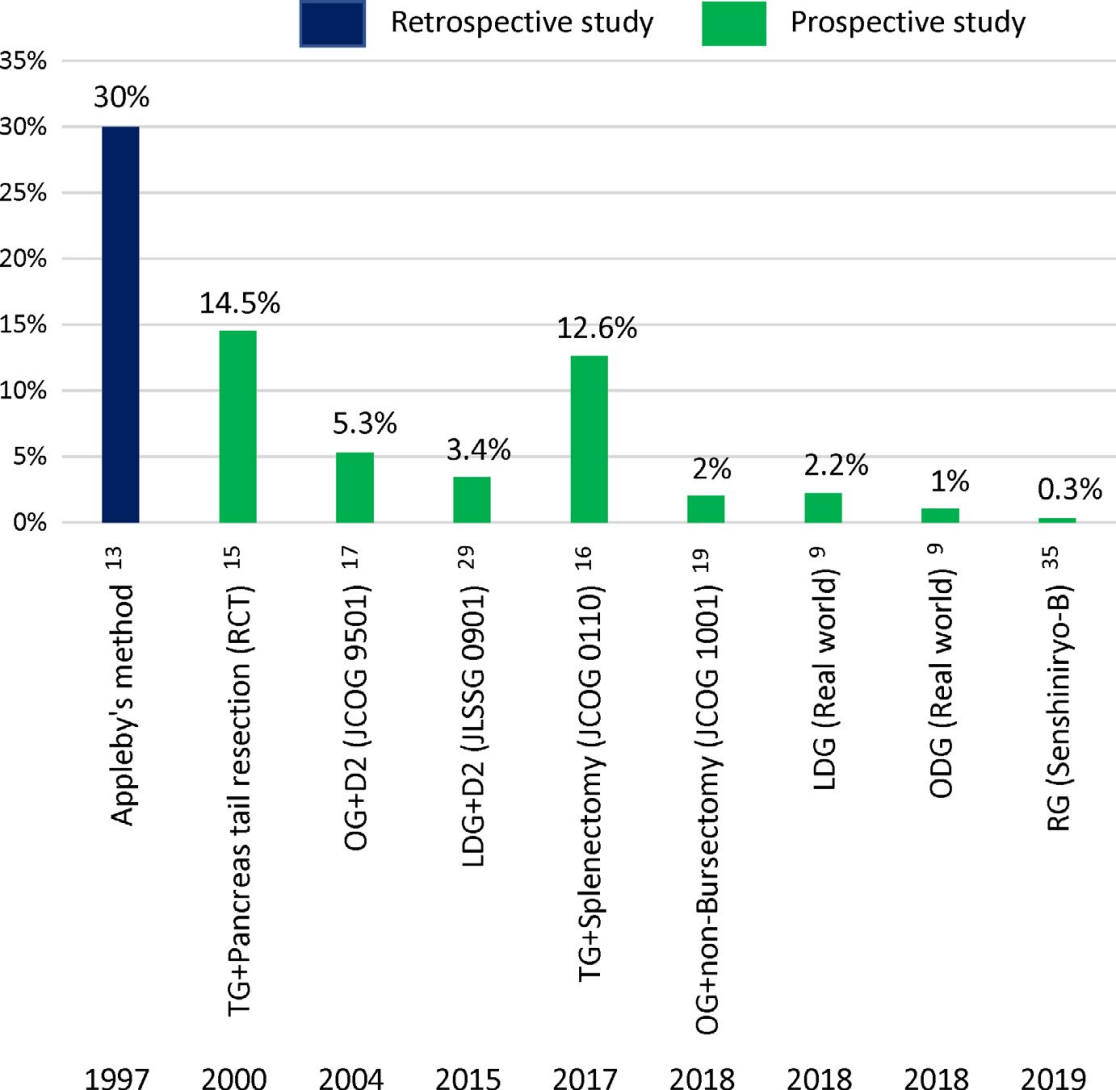
Incidence of postoperative pancreatic fistula after gastrectomy.^9^, ^13^, ^15^, ^16^, ^17^, ^19^, ^29^, ^34^ Green bars indicate prospective studies, and dark blue bar indicates a retrospective study. The rate of postoperative pancreatic fistula incidence was reported as 30% after open gastrectomy (OG) with pancreaticosplenectomy in 1997,^13^ but lately has improved to below 1% with robotic gastrectomies (RGs) in 2019.^32^ TG, total gastrectomy; RCT, randomized controlled trials; DG, distal gastrectomy; LDG, laparoscopic distal gastrectomy; ODG, open distal gastrectomy

Earlier reports regarding the incidence of postoperative pancreatic fistula described higher rates in more invasive gastrectomies than the current standard. An incidence of 30% was reported for the Appleby operation,^13^ while rates of 15.2% and 14.5% have been reported for gastrectomy plus splenectomy with concurrent pancreatic resection in a retrospective study^14^ and a prospective cohort study,^15^ respectively. Thus, concurrent pancreatic resection has been considered to carry the highest risk for postoperative pancreatic fistula in gastrectomy. On the other hand, total gastrectomy (TG) with splenectomy but no resection of the pancreas showed a 12.6% incidence of postoperative pancreatic fistula in the Japanese Clinical Oncology Group (JCOG) trial, JCOG0110, which explored the prognostic benefit of splenectomy in TG for proximal gastric cancer of T2‐4/N0‐2/M0 not invading the greater curvature^16^ (Figure 1). These clinical outcomes suggest that splenectomy also carries a high risk for postoperative pancreatic fistula after D2 gastrectomy.

In contrast, standard gastrectomy (with bursectomy) with D2 lymph node dissection showed a postoperative pancreatic fistula frequency of around 6% in the JCOG9501 trial, a prospective study conducted in 2004 by the JCOG for Gastric Cancer.^17^ These earlier reports represented intuitive frequencies of postoperative pancreatic fistula in OG for advanced gastric cancer, because there was no standard definition of postoperative pancreatic fistula, and such old reports did not refer to the grade of severity according to the Clavien‐Dindo (CD) classification.^18^ The most recent clinical trial, JCOG1001, conducted in 2018, used the CD classification, and intriguingly also showed a similar postoperative pancreatic fistula frequency of 5% for CD grade III, in contrast to no bursectomy (2%; *P* = 0.032).^19^ This postoperative pancreatic fistula rate reported in 2018 reconfirmed the results of the previous cohort reported in Japan in 2004.^17^ Postoperative pancreatic fistula described in the early reports is sure to be similar to CD grade III postoperative pancreatic fistula.

During open TG, patients often need supra‐pancreatic lymph node dissection of No.11d, 4sa, and 10, together with splenectomy. It is well known that the incidence of postoperative pancreatic fistula is increased in these procedures due to splenectomy. In fact, it was reported that the postoperative pancreatic fistula incidence of TG with splenectomy was 12.6% in the JCOG0110 trial,^16^ while the postoperative pancreatic fistula incidence of distal gastrectomy (DG)+D2 was 5.3% in the JCOG9501 trial.^17^ Therefore, postoperative pancreatic fistula after open TG is more likely than after open DG and careful attention should be paid to this point. The incidence of postoperative pancreatic fistula after gastrectomy in advanced gastric cancer in Japan is listed in Table 1.

**Table 1:** Incidence of postoperative pancreatic fistula after open gastrectomy for gastric cancer

Author, Year	Incidence	Procedure	Type of trial	Patients	Country
Furukawa, 1997^13^	30.0%	Left upper abdominal exenteration + Appleby's method for type 4 AGC	Single‐institution retrospective study	54	Japan
Otsuji, 1999^14^	15.2%	TG + pancreaticosplenectomy	Single‐institution retrospective study	128	Japan
Furukawa, 2000^15^	14.5%	TG + pancreas tail resection	RCT	110	Japan
Sano, 2016^16^	12.6%	TG + splenectomy	RCT (JCOG0110)	505	Japan
Sano, 2004^17^	5.3%	OG + D2 alone	RCT (JCOG9501)	523	Japan
	6.2%	OG + D2+PAND			
Kurokawa, 2018^19^	5.0%	OG + bursectomy	RCT (JCOG1001)	1204	Japan
	2.0%	OG + omentectomy (no bursectomy)			
Bonenkamp, 1995^3^	3.0%	Gastrectomy + D2	RCT (Dutch trial)	711	Netherlands

Abbreviations: AGC, advanced gastric cancer; OG, open gastrectomy; PAND, para‐aortic nodal dissection; RCT, randomized control trial; TG, total gastrectomy.

In the Western world, the frequency of postoperative pancreatic fistula was recognized to be 3% in gastrectomy with D2 lymph node dissection in a Dutch trial that was supervised by the Japanese expert surgeon, Sasako.^3^ There may be a number of possible reasons why the postoperative pancreatic fistula incidence was low in this representative European clinical trial (Dutch trial). First, when the Dutch trial was reported, the concept of pancreatic fistula had not been established and there was no clear definition of the condition. Therefore, it is difficult to compare the results of trials between both Western and Eastern worlds, because they were evaluated using different standards for distinct clinics, unlike later trials where evaluation has been based on the common established criteria. Secondly, pancreatic fistula may have been more frequently recognized in Japan where the concept of supra‐pancreatic node dissection had been well‐established as a rigorous surgery (D2 lymph node dissection with bursectomy) for advanced gastric cancer. For example, it is said that pancreatic fistula is more likely to occur when the pancreatic capsule is removed in procedures, such as bursectomy, which is considered as the main reason why the JCOG1001 study also shown a high incidence of postoperative pancreatic fistula. Postoperative pancreatic fistula may be less likely to occur in Europe because bursectomies are rarely performed there.

Recently, in a neoadjuvant chemotherapy (NAC) setting, the postoperative pancreatic fistula incidence was reported as 2% after gastrectomy for serosa‐positive gastric cancer with preoperative doublet NAC of CDDP+S1 in the JACCRO‐GC01 trial.^20^ Moreover, the KDOG1001 trial reported a postoperative pancreatic fistula incidence of 12.5% after gastrectomy for giant type III/IV gastric cancer with preoperative triplet NAC of docetaxel+CDDP+S1.^21^ It has not been demonstrated that NAC increases postoperative pancreatic fistula rates after gastrectomy. Indeed, postoperative pancreatic fistula development seems to be related to a number of factors, such as advanced degree of gastric cancer, chemotherapy regimen, and operative procedure. Moreover, Yoshikawa et al noted in the JACCRO GC‐01 study that postoperative pancreatic fistula‐induced intra‐abdominal abscess may be counted as postoperative intra‐abdominal abscess. Since clinical trials for post NAC gastrectomy have limited target cases, the number of registered patients is often relatively small, and so even a difference of one patient is likely to have an effect on incidence rates. This may be one of the reasons for the variability in postoperative pancreatic fistula incidence rates in post NAC gastrectomy reports.

### 2.2 Laparoscopic gastrectomy

The recent prevalence of LG for both early and advanced gastric cancer is actively reported, and its clinical outcomes for EGC with limited lymphadenectomy have been initially disclosed. Laparoscopic surgery has the disadvantage that the operating angle and field of view of surgical instruments are limited. Since the area around the pancreas is anatomically complicated and lymph node dissection is difficult, there is a high possibility that the pancreas will be injured due to the limited range of motion of the instruments. Therefore, pancreatic fistula is considered to be a complication that can be uniquely found in laparoscopic surgery, rather than in a conventional laparotomy, and it could greatly affect the patient's life and/or significantly extend the length of hospital stay. For these reasons, postoperative pancreatic fistula has been evaluated as a critical complication which may be unique in laparoscopic surgery in comparison to open surgery.

The first Japanese large‐scale multi‐institutional phase II trial exploring the safety of LDG for patients with clinical stage I gastric cancer, JCOG0703, showed the incidence of postoperative pancreatic fistula after gastrectomy was 1.1%.^22^ Several similar studies also showed the incidence of postoperative pancreatic fistula as 0.5%‐5%.^23^, ^24^, ^25^ In addition, JCOG1401 was the first prospective study to evaluate the safety of proximal/total gastrectomy for clinical stage I proximal gastric cancer.^26^ In this trial, the incidence of postoperative pancreatic fistula was 2%, in contrast to previous reports of EGC treated by open surgery that disclosed rates of 4%^27^ to 15%^28^ for TG.

Clinical outcomes of LG (bursectomy not mandatory) were reported as 3.4% for advanced gastric cancer (T2 or beyond) in DG with D2 lymph node dissection in the JLSSG0901 trial.^29^ Other countries in Asia have also reported the results of RCTs comparing the outcomes of LG and OG for advanced gastric cancer. The rates of postoperative pancreatic fistula after LDG were 0.4% in the CLASS‐01 trial conducted in China^30^ and 1.9% in the KLASS‐02 trial conducted in Korea.^31^

Furthermore, Uyama from Fujita Health University has had rigorous experience (including splenectomy with concurrent pancreatic resection) of laparoscopic TG for advanced gastric cancer, and reported a total rate of 12% for pancreatic fistula of CD grade III or higher in a single‐institute experience in Japan.^32^

Finally, robotic gastrectomy using multi‐jointed devices for gastric cancer, including advanced tumors, is likely to be beneficial for reducing the postoperative pancreatic fistula incidence, because recent literature shows the incidence of postoperative pancreatic fistula with robotic gastrectomy to be 0% (0/521)^33^, 0.9%,^34^

and 0.3%^35^ in Japan and 0%^36^ in other countries. The incidence of postoperative pancreatic fistula after LG in gastric cancer is listed in Table 2.

**Table 2:** Incidence of postoperative pancreatic fistula after laparoscopic gastrectomy

Author, Year	Incidence	Procedure	Type of trial	Patients	Country
Katai, 2010^22^	1.1%	LDG for stage I GC	Multi‐institution prospective study (JCOG0703)	176	Japan
Yoshikawa, 2013^23^	0.5%	LDG for stage I GC	Prospective study	193	Japan
Wada, 2014^24^	5.0%	LTG for stage I GC	Single‐institution retrospective study	100	Japan
Kawamura, 2015^25^	5.6%	LTG + D2+splenectomy	Single‐institution retrospective study	259	Japan
Katai, 2019^26^	2.0%	LTG, LPG for stage I GC	Multi‐institution prospective study (JCOG1401)	244	Japan
Inaki, 2015^29^	3.4%	LDG + D2 for AGC	RCT (JLSSG0901)	180	Japan
Nakauchi, 2016^32^	12.0%	LTG for AGC	Single‐institution retrospective study	92	Japan
Nakauchi, 2016^33^	0.0%	RG	Single‐institution retrospective study	521	Japan
Okabe, 2019^34^	0.9%	RG	Multi‐institution prospective study	115	Japan
Uyama, 2019^35^	0.3%	RG	Multi‐institution prospective study (Advanced Medical Technology “Senshiniryo” B.)	326	Japan
Kun Yang, 2019^36^	0.0%	Robotic spleen‐preserving splenic hilar lymphadenectomy	Single‐institution retrospective study	93	Korea

Abbreviations: AGC, advanced gastric cancer; GC, gastric cancer; LDG, laparoscopic distal gastrectomy; LPG, laparoscopic proximal gastrectomy; LTG, laparoscopic total gastrectomy; RCT, randomized control trial; RG, robotic gastrectomy.

## 3 DIAGNOSIS OF POSTOPERATIVE PANCREATIC FISTULA

Postoperative pancreatic fistula mainly represents a parenchymal leak not directly related to a pancreatic‐enteric anastomosis after gastrectomy, different to pancreatectomy.^37^ The objective diagnosis of postoperative pancreatic fistula is difficult because it is often confused with intra‐abdominal abscess due to the similarity of their clinical features. The International Study Group definition of pancreatic fistula (ISGPF) defines postoperative pancreatic fistula as an increased drain amylase concentration of greater than three times the serum amylase activity at postoperative day 3, and has classified postoperative pancreatic fistula into three categories: A, B, and C (Table 3).

**Table 3:** Parameters for postoperative pancreatic fistula grading (ISGPF)

Grade	A	B	C
Clinical conditions	Well	Often well	Ill appearing/bad
Specific treatment^a^	No	Yes/no	Yes
US/CT (if obtained)	Negative	Negative/positive	Positive
Persistent drainage (after 3 wk)^b^	No	Usually yes	Yes
Reoperation	No	No	Yes
Death related to POPF	No	No	Possibly yes
Signs of infections	No	Yes	Yes
Sepsis	No	No	Yes
Readmission	No	Yes/no	Yes/no

Abbreviations: CT, computed tomography; ISGPF, International study group definition of pancreatic fistula; POPF, postoperative pancreatic fistula; US, ultrasonography.

^a^ Partial (peripheral) or total parenteral nutrition, antibiotics, enteral nutrition, somatostatin analogue and/or minimal invasive drainage.

^b^ With or without a drain in situ.

Recent studies, however, have favored the CD grading classification over the ISGPF grading classification for postoperative pancreatic fistula assessment. The CD classification grading is very simple, in which grade III requires surgical, endoscopic, or radiological intervention, in contrast to grade I/II which only require pharmacological treatment at most (Table 4).^18^ The incidence of postoperative pancreatic fistula prior to the use of the CD classification^17^ was almost the same as that defined by CD grading in the JCOG trials,^19^ meaning that objective CD grading confirms the expert surgeon's recognition of postoperative pancreatic fistula.

**Table 4:** Parameters for postoperative pancreatic fistula grading (Clavien‐Dindo Classification of Surgical Complications)

Grade	Definition
Grade I	Any deviation from the normal postoperative course without the need for pharmacological treatment or surgical, endoscopic, and radiological interventions. Allowed therapeutic regimens are: drugs as antiemetics, antipyretics, analgetics, diuretics, electrolytes, and physiotherapy. This grade also includes wound infections opened at the bedside.
Grade II	Requiring pharmacological treatment with drugs other than such allowed for grade I complications. Blood transfusions and total parenteral nutrition are also included.
Grade III	Requiring surgical, endoscopic or radiological intervention.
Grade IIIa	Intervention not under general anesthesia.
Grade IIIb	Intervention under general anesthesia.
Grade IV	Life‐threatening complication (including CNS complications)^a^ requiring IC/ICU management.
Grade IVa	Single organ dysfunction (including dialysis).
Grade IVb	Multiorgan dysfunction.
Grade V	Death of a patient.

Abbreviations: CNS, central nervous system; IC, intensive care; ICU, intensive care unit.

^a^ Brain hemorrhage, ischemic stroke, subarrachnoidal bleeding, but excluding transient ischemic attacks.

Initial attention in predicting postoperative pancreatic fistula occurred with TG. Sano et al first discovered that drain amylase concentration at postoperative day 1 is a simple and useful marker for the prediction of postoperative pancreatic fistula in TG; eight of 13 patients with postoperative pancreatic fistula (62% diagnostic sensitivity) showed high drain amylase concentrations (4000 U/L or higher), while 63 of 82 patients with no postoperative pancreatic fistula (77% diagnostic specificity) showed low drain amylase concentrations.^38^ Miki et al also reported that the most optimal cut‐off value of drain amylase concentration at postoperative day 1 was 3398 U/L to predict ISGPF grade B/C in TG, with a diagnostic sensitivity of 65%, and specificity of 77%.^39^ Intriguingly, objective ISGPF grading also confirms the expert surgeon's recognition of postoperative pancreatic fistula.

Tomimaru et al reported that diagnostic drainage inspection with a dark red colored drainage fluid at postoperative day 1 is sufficient to predict postoperative pancreatic fistula (ISGPF grade B/C) in comparison to drain amylase concentration in TG. With drain amylase concentration (5000 U/L or higher), diagnostic sensitivity was 100% and diagnostic specificity was 82%,^40^ while with drain inspection, diagnostic sensitivity was 100% and diagnostic specificity was 78%.

Kanda et al explored the most optimal cut‐off value of drain amylase concentration in laparotomy‐assisted gastrectomy limited to DG to predict ISGPF grade B/C, and found it to be 4078 U/L with a sensitivity of 0.75 and specificity of 0.86. More interestingly, a combination with C‐reactive protein values at postoperative day 3 increased the prediction sensitivity of postoperative pancreatic fistula.^41^ These findings suggest that the optimal cut‐off value of drain amylase concentration is not so different between TG and DG.

More recently, the optimal cut‐off value was evaluated in patients who underwent curative gastrectomy. Taniguchi et al examined 591 gastric cancers to predict ISGPF grade B/C postoperative pancreatic fistula and determined the most predictable indicator to be a drain amylase concentration of 2100 U/L at postoperative day 3; diagnostic sensitivity was 83%, while diagnostic specificity was 99%.^42^ This is the first report describing the clinical utility of drain amylase concentration at postoperative day 3.

Since then, Kamiya et al reported that a two‐point measurement of drain amylase concentration at both postoperative days 1 and 3 could more precisely predict severe postoperative pancreatic fistula (CD III) after gastrectomy.^43^ They reported that the diagnostic sensitivity of the two‐point measurement was 65% with a 90% specificity, while the sensitivities of the day 1 and day 3 measurements were 73% and 75%, with specificities of 83% and 79%, respectively. They found the optimal cut‐off values of drain amylase concentration to be 2218 U/L and 555 U/L at postoperative day 1 and 3, respectively.

The remarkable differences in the optimal cut‐off values among the independent studies is of concern at the present time, but may be derived from the differential diagnosis of postoperative pancreatic fistula (ISGPF or CD), the type of operative procedures, or complication rates. Optimal standardization of the drain amylase concentration would be anticipated for the prediction of postoperative pancreatic fistula in the near future.

## 4 RISK OF POSTOPERATIVE PANCREATIC FISTULA

Although risk of postoperative pancreatic fistula is due to invasive operative procedures as shown in Figure 1, other clinical factors can also affect its risk after gastrectomy. Being overweight (high body mass index [BMI]) is a reproducible risk factor for postoperative pancreatic fistula. The JCOG9501 trial confirmed for the first time that a high BMI increased the risk of surgical complications, including postoperative pancreatic fistula, in patients undergoing a D2 dissection,^44^ and various similar indicators representing being overweight have been rigorously proposed, such as BMI,^32^, ^34^, ^45^, ^46^ visceral fat area,^47^ and fatty pancreas.^48^ Jiang et al identified male sex as an independent predictor for postoperative pancreatic fistula as well as BMI in a multivariate analysis.^46^ This may be due to difficulty in tissue handling and the vast area of visceral fat. Recently, in the Japanese national clinical database (NCD) which includes 39 253 cases with TG, male sex, splenectomy, and Brinkman index were selected as common risk factors for postoperative pancreatic fistula.^49^ Tobacco contains a large number of different toxic substances, including nicotine, and is known to cause inflammation, endothelial impairment, and thrombus formation.^50^ It is believed that tissue ischemia results in delayed wound healing after surgery, increasing complications. Additionally, several epidemiologic studies uncovered the independent effects of tobacco smoking on the development of chronic pancreatitis.^51^

Although the direct mechanism by which smoking causes postoperative pancreatic fistula after gastrectomy has not been clarified, an association between smoking and pancreatic disorders has been reported, and smoking may contribute to pancreatic fistula after gastrectomy.

Other groups proposed that the pancreas shape is also a risk factor for postoperative pancreatic fistula, due to factors such as any protruding tissue (process) of the pancreas head,^52^ the length between the levels of the pancreatic body surface and the root of the common hepatic artery,^53^ pancreatic position defined by pancreas‐aorta length, and the angle between the aorta and celiac artery.^54^ Recent advancement of imaging technology can therefore help surgeons carefully treat the pancreas during surgery.

**Figure 2:**
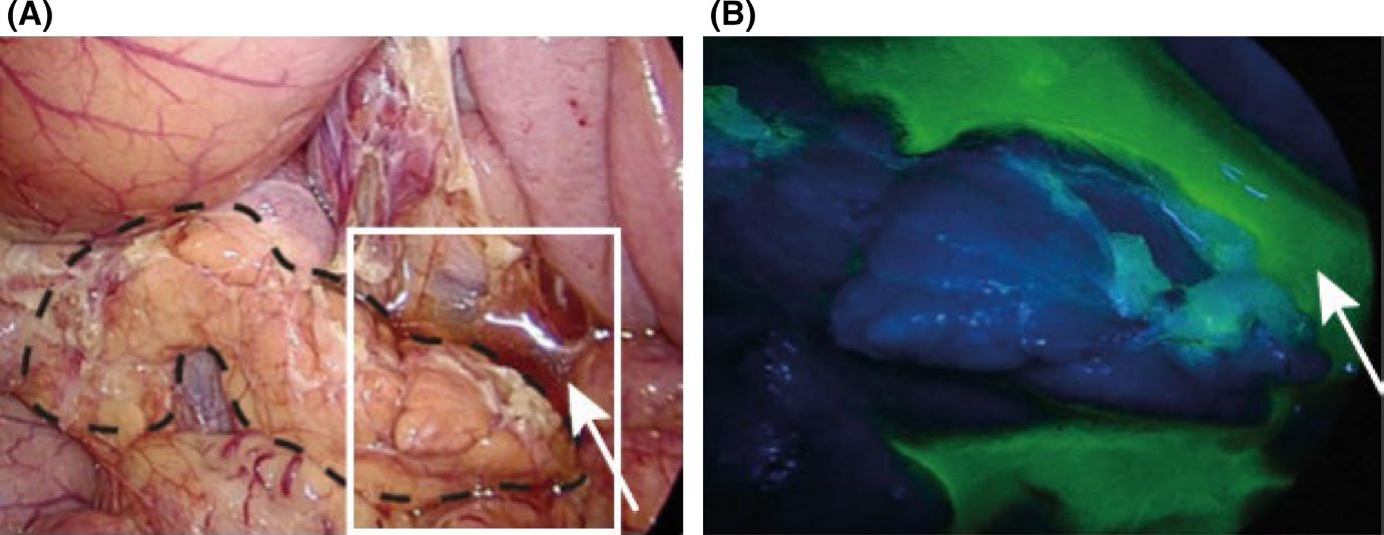
Fluorescent imaging of the pancreas by a chymotrypsin probe. Gross appearance in natural color (A). Image obtained through light‐blocking glasses 2 minutes after administration of the chymotrypsin probe (B). The black dotted line in (A) indicates the borders of the pancreas. The white arrow in (B) indicates ascites containing pancreatic juice. These figures were cited from Ida et al (2018)^55^

Risk factors for postoperative pancreatic fistula due to intraoperative manipulation include pressure and thermal injury to the pancreas. Ida et al investigated pressure injury by using blunt trauma to compress the pancreas during laparoscopic surgery on pigs, and showed that it could result in pancreatic juice leakage. The pancreas of pigs were gently compressed dorsally for 15 minutes laparoscopically with gauze grasped with forceps, and pancreatic juice leakage was visualized by fluorescence imaging after topical administration of chymotrypsin‐activatable fluorophore in real time (Figure 2). Amylase concentrations were highly elevated after the procedures, and the pancreas showed necrotic histological change.^55^ This experiment suggested that compressing the pancreas during LG should be of major concern, and should be avoided in order to prevent postoperative pancreatic fistula. Another risk, thermal injury, may result from the use of energy devices. Pogorelić et al conducted an animal experiment and demonstrated that the use of ultrasonic coagulation and dissection equipment at high power for extended periods of time causes lateral heat damage.^56^ Irino et al experimented with swine pancreas and reported that as the number of harmonic scalpel activations increased, the temperature of the pancreatic surface in front of the tip of the active blade increased. Histologically, only serosal damage was observed after three activations, whereas thermal damage to the pancreatic body occurred after five or seven activations of the harmonic scalpel.^57^

We speculate that there are specific circumstances unique to LG that may be involved in the frequent occurrence of postoperative pancreatic fistula in LG. The first point is that strong pressure with forceps concentrates the force to one specific point on the tip of the forceps in laparoscopic surgery. The second point is that the leverage principle works as the port is used as a fulcrum, and the forceps will be pushed with a stronger force than expected by the operators. On the other hand, manual compression in laparotomy distributes the force over the entire push area of the hand. In addition, since the organ is touched directly from a close location during laparotomy, it is easier to fine tune the applied force in this way than via a laparoscope.

## 5 PREVENTION OF POSTOPERATIVE PANCREATIC FISTULA

Obama et al first reported that drain amylase levels were higher in LG than in OG.^58^ Allowing for this finding, Hiki et al demonstrated that LDG (2.2%) was more often associated with grade B or higher postoperative pancreatic fistula than OG (1.0%; *P* = 0.04) after propensity patient matching in the prospective Japanese NCD, which included 5288 patients in 2014.^9^ The NCD reinforced a similar result (1.0% in LDG versus 0.8% in open DG, *P* = 0.01).^59^ These data suggest that gastric cancer surgeons must be very careful to prevent postoperative pancreatic fistula in LG.

As a measure against the compression issues during LG, Tsujiura et al proposed “pancreas compressionless gastrectomy,” where the amylase concentrations from the drain tube in the compressionless group were significantly lower on postoperative days 1 and 3 (*P* < 0.001 and *P* = 0.013, respectively) compared with the compression group.^60^ The rates of severe postoperative pancreatic fistula and intra‐abdominal infectious complications decreased from 11.8% to 2.2% (*P* = 0.116) and from 17.6% to 2.2% (*P* = 0.018), respectively. This report also supported Migita's hypothesis that postoperative pancreatic fistula increases in patients with a specific anatomical condition as shown on computed tomography imaging, such as a long length between the pancreatic body surface and the root of the common hepatic artery, results in intraoperative pancreatic compression.^53^

**Figure 3:**
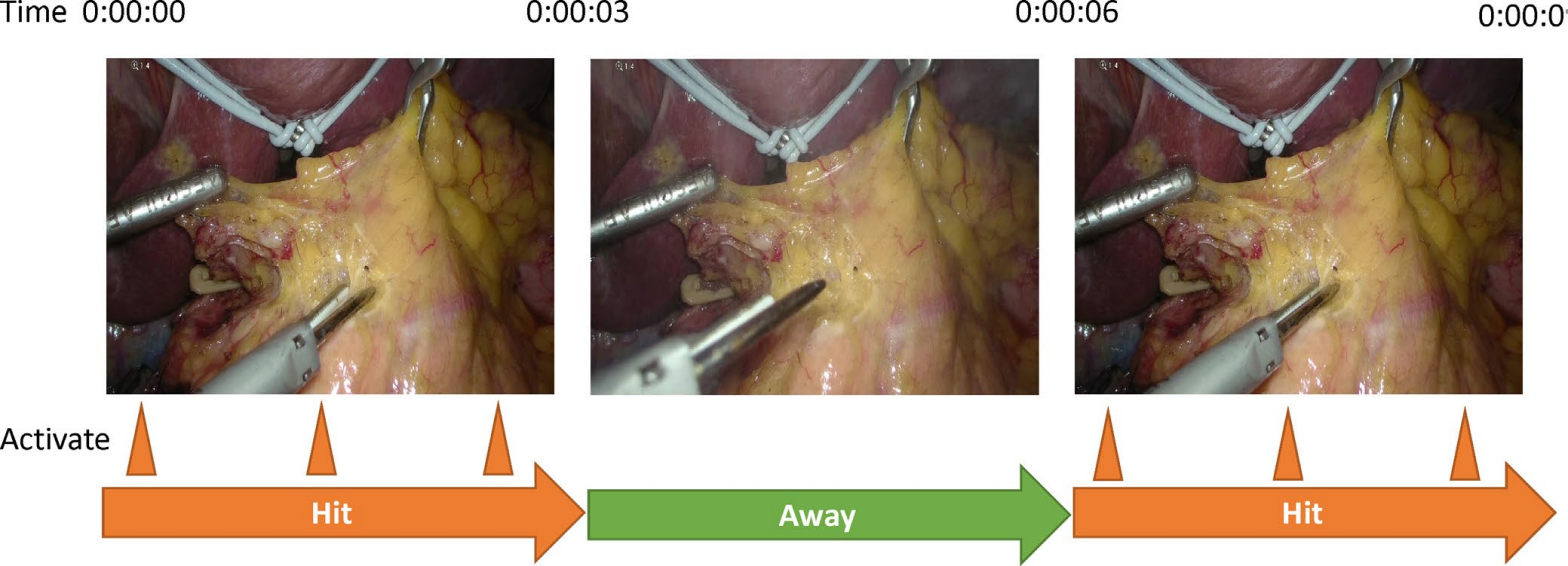
‘Hit and Away’ technique. In the ‘‘Hit’’ phase, surgeons perform three activations with the tip of the ultrasonic scalpel after the tissues and vessels are clamped in a block. After three activations, the ultrasonic scalpel is immediately released. These figures were cited from Irino et al (2016)^57^

Emerging techniques, from alternate approaches to gently handling the pancreas, might provide a remarkable reduction in the incidence of postoperative pancreatic fistula. For example, the “Hit and Away” technique is an advanced procedure that could reduce thermal injury to the pancreas and further reduce postoperative pancreatic fistula.^57^ In an animal experiment, the tissue temperature of swine mesocolon reached 43°C, a temperature at which adipose tissue melted but fibrous tissue including vessels remained intact. The temperature returned to baseline within 3 seconds of turning off the ultrasonic scalpel, proposing the advantage of using ultrasonic scalpels in a pulsatile manner. So, the “Hit and Away” technique applies this pulsatile usage of an ultrasonic scalpel to reduce postoperative pancreatic fistula (Figure 3).

## 6 CONCLUSION

We extensively reviewed the current understanding of postoperative pancreatic fistula after gastrectomy, exploring its incidence, definition for diagnosis, prediction prior to severe clinical outcomes, and surgical risk and prevention. Importantly, manipulation of the pancreas should be minimized to reduce postoperative pancreatic fistula incidence if oncological matters can permit. Moreover, even indirect compression of the pancreas and even slight thermal injury can have significant risks for postoperative pancreatic fistula during LG. Thus, there is a need for improvement in surgical techniques.

## DISCLOSURE

Conflict of Interest: Authors declare no conflicts of interest for this article.
